# Publisher Correction: Methylation deficiency disrupts biological rhythms from bacteria to humans

**DOI:** 10.1038/s42003-020-1031-0

**Published:** 2020-06-04

**Authors:** Jean-Michel Fustin, Shiqi Ye, Christin Rakers, Kensuke Kaneko, Kazuki Fukumoto, Mayu Yamano, Marijke Versteven, Ellen Grünewald, Samantha J. Cargill, T. Katherine Tamai, Yao Xu, Maria Luísa Jabbur, Rika Kojima, Melisa L. Lamberti, Kumiko Yoshioka-Kobayashi, David Whitmore, Stephanie Tammam, P. Lynne Howell, Ryoichiro Kageyama, Takuya Matsuo, Ralf Stanewsky, Diego A. Golombek, Carl Hirschie Johnson, Hideaki Kakeya, Gerben van Ooijen, Hitoshi Okamura

**Affiliations:** 1 Graduate School of Pharmaceutical Sciences, Laboratory of Molecular Metabology, Kyoto University, Kyoto, Japan; 2 Graduate School of Pharmaceutical Sciences, Kyoto University, Kyoto, Japan; 3 Graduate School of Pharmaceutical Sciences, Department of System Chemotherapy and Molecular Sciences, Kyoto University, Kyoto, Japan; 4 Institute of Neuro- and Behavioral Biology, University of Münster, Münster, Germany; 5 School of Biological Sciences, University of Edinburgh, Edinburgh, UK; 6 Department of Psychiatry and Biobehavioral Sciences, University of California, Los Angeles, Los Angeles, CA USA; 7 Department of Biological Sciences, Vanderbilt University, Nashville, TN USA; 8 Karolinska Institute, Stockholm, Sweden; 9 Department of Science and Technology, National University of Quilmes/CONICET, Buenos Aires, Argentina; 10 Institute for Frontier Life and Medical Sciences, Kyoto University, Kyoto, Japan; 11 Centre for Cell and Molecular Dynamics, Department of Cell and Developmental Biology, University College London, London, UK; 12 Molecular Medicine, Peter Gilgan Centre for Research and Learning (PGCRL), The Hospital for Sick Children, Toronto, ON Canada; 13 Department of Biochemistry, University of Toronto, Toronto, ON Canada; 14 Center for Gene Research, Nagoya University, Nagoya, Japan; 15 Graduate School of Pharmaceutical Sciences, Laboratory of Molecular Brain Science, Kyoto University, Kyoto, Japan; 16 Present Address: The University of Manchester, Faculty of Biology, Medicine and Health, Oxford Road, Manchester, M13 9PL UK; 17 Present Address: Kyoto University, Graduate School of Medicine, Department of Neuroscience, Division of Physiology and Neurobiology, Yoshida-Konoe-cho, Sakyo-ku, Kyoto, 606-8501 Japan

**Keywords:** Circadian rhythms, Evolution, Metabolism

Correction to: *Communications Biology* 10.1038/s42003-020-0942-0, published online 06 May 2020.

The original version of this Article contained an error in the author affiliations.

Affiliation 17 incorrectly read ‘Present address: Kyoto University, Graduate School of Medicine, Department of Neuroscience, Division of Physiology and Neurobiology, Yoshida-Konoe-cho, Oxford Road, Sakyo-ku, Kyoto 606-8501, Japan.’

This has now been corrected in both the PDF and HTML versions of the Article.

