# Supplementary material for: Methylation deficiency disrupts biological rhythms from bacteria to humans
Source: Commun Biol. 2020 May 6;3:211. doi: 10.1038/s42003-020-0942-0 (PMC7203018; doi:10.1038/s42003-020-0942-0)
Supplement: Supplementary file 1 — Supplementary Information [file 42003_2020_942_MOESM1_ESM.pdf]

## SUPPLEMENTARY FIGURES AND LEGENDS

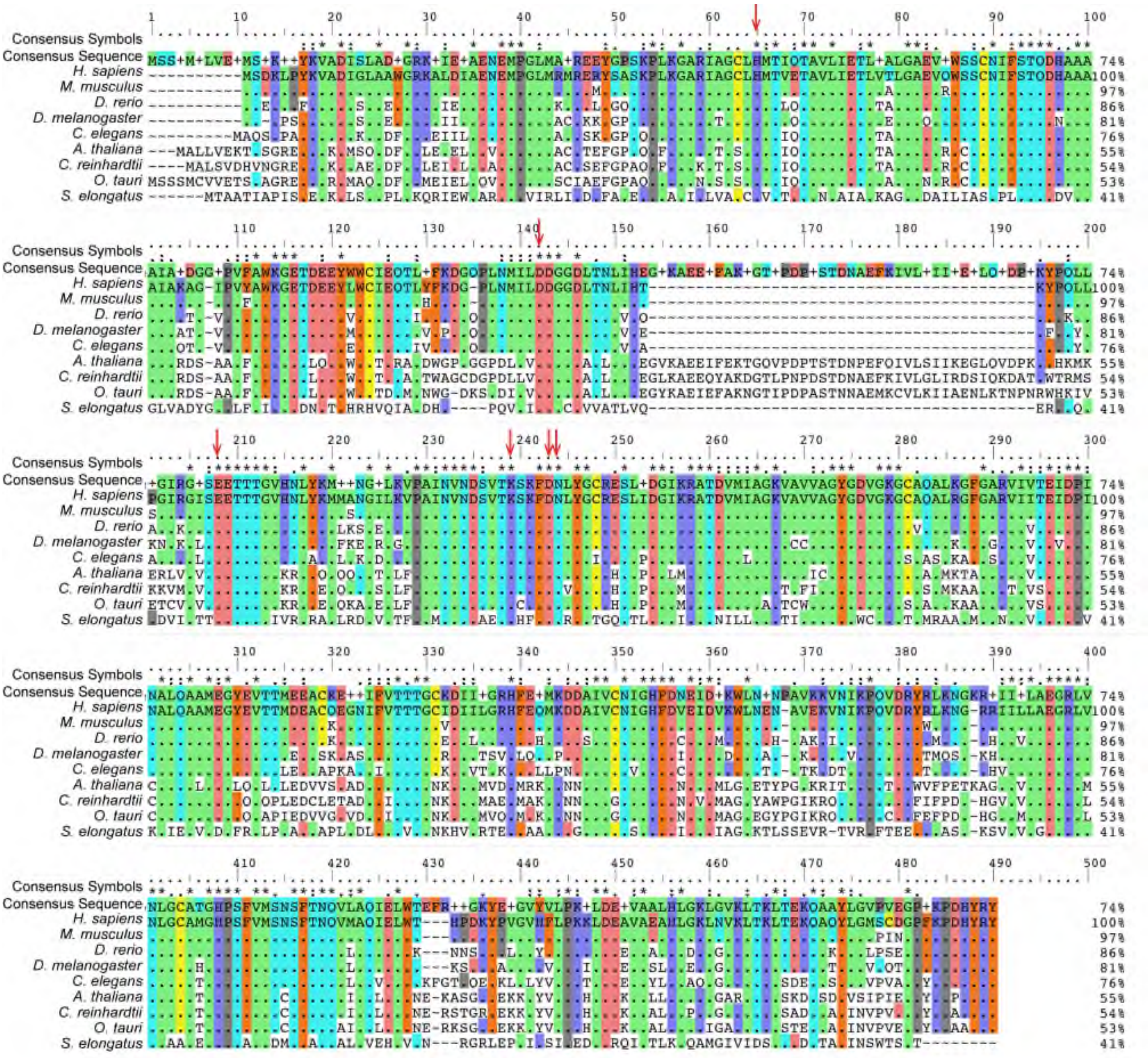


**Supplementary Figure 1: Sequence alignment of full-length AHCY.**

When amino acids are identical to human, a dot is shown in the alignment. Sequence identities (%), relative to human AHCY, are given on the right. The consensus symbols are shown on top (* = fully conserved residue, : = conservation of strongly similar properties, . = conservation of weakly similar properties). Red arrows indicate the residues that are essential for rat AHCY function. The source of sequences is, for human, Genbank: NP_000678.1; mouse, Genbank: NP_057870.3; zebrafish, Genbank: NP_954688.1; fruit fly, Genbank: NP_511164.2; *C. elegans*, Genbank: NP_491955.1; *Arabidopsis*: NP_193130.1; *Chlamydomonas*, Genbank: XP_001693339.1; *Ostreococcus*, Genbank: XP_022839640.1; cyanobacteria, Genbank: WP_011243218.1. Related to Figure 1.

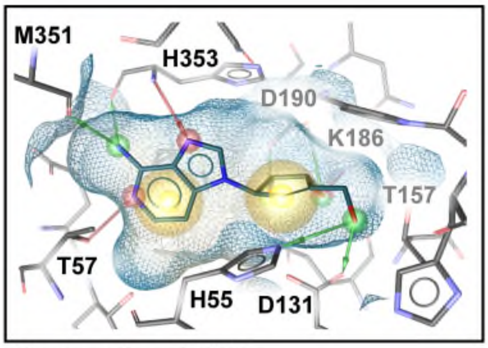

*M. musculus* (5AXA)

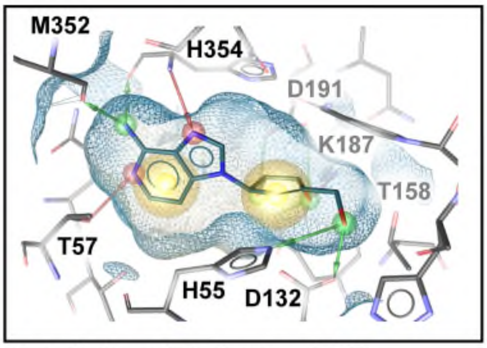

*D. rerio* (template human 1LI4)

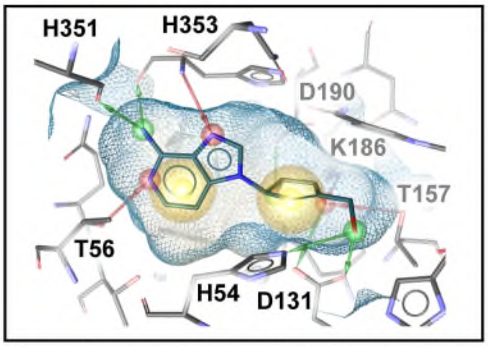

*D. melanogaster* (template human 1LI4)

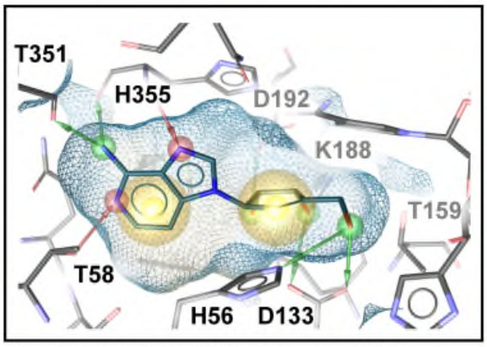

*C. elegans* (template human 1LI4)

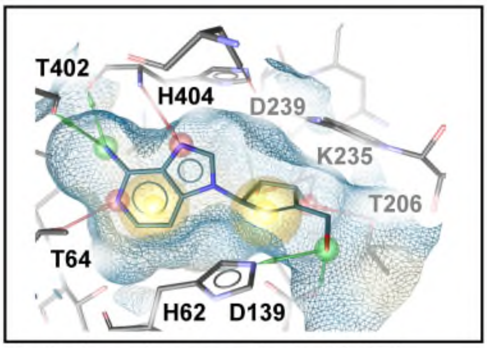

*A. thaliana* (template lupin 3OND)

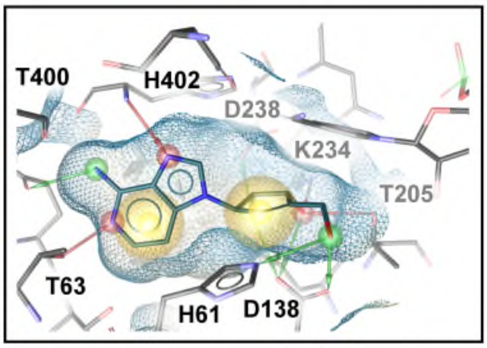

*C. reinhardtii* (template lupin 3OND)

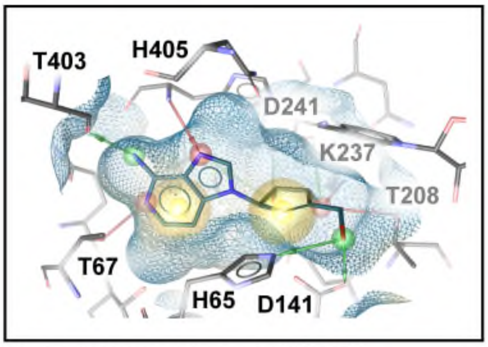

*O. tauri* (template lupin 3OND)

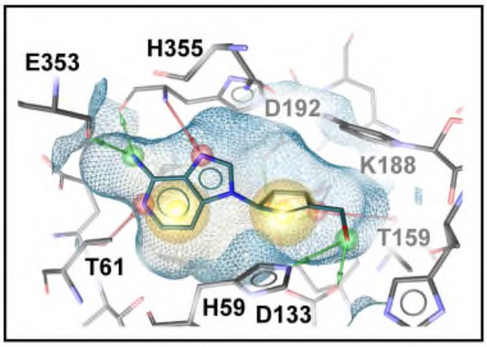

*S. elongatus* (template human 1LI4)

**Supplementary Figure 2: Molecular docking simulations of AHCY with DZnep.**

Docking simulations of AHCY with DZnep, for each species as indicated below each picture, based on a published template crystal structure also indicated. The amino acids involved in DZnep binding are indicated together with their position. Note their conservation. The M351 in mouse (or human, see Fig. 1) is involved in DZnep binding via its backbone, explaining why, although this residue appears at first not conserved (H in fly, T in worm, plants and algae, and E in cyanobacteria), its conserved position in the active site is important. Red and green arrows are hydrogen bonds, yellow spheres are hydrophobic effects. Hydrogen atoms are not shown. The estimated free energies of binding for depicted DZnep docking conformations in kcal/mol were -9.92 for *M. musculus*, -9.50 for *D. rerio*, -9.29 for *D. melanogaster*, -9.46 for *C. elegans*, -9.50 for *A. thaliana*, -9.26 for *C. reinhardtii*, -9.68 for *O. tauri*, and -9.43 for *S. elongatus*. Related to Figure 1.

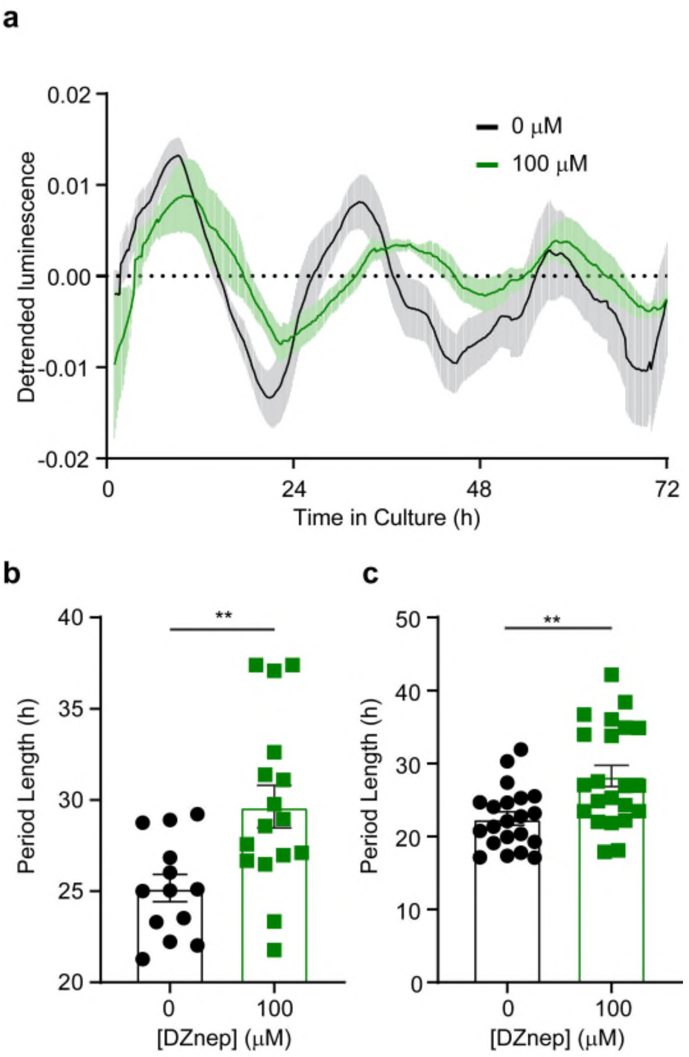


**Supplementary Figure 3: The link between the methyl cycle and the clock is conserved in invertebrates.**

(**a**), Mean luminescence ± SEM of freely moving *Caenorhabditis elegans* populations of ~100 nematodes treated with vehicle (n = 10 populations) or 100 μM DZnep (n = 6 populations). (**b**), Mean period ± SEM of independent populations, compared by Student *t*-test ; **, $p < 0.01$; n = 13 populations treated with vehicle and n = 16 populations treated with 100 μM DZnep. (**c**) Mean period ± SEM obtained from single nematodes in isolation, n = 21 for controls and 22 for 100 μM DZnep, analyzed by Student *t*-test, **, $p < 0.01$. Related to Figure 2.

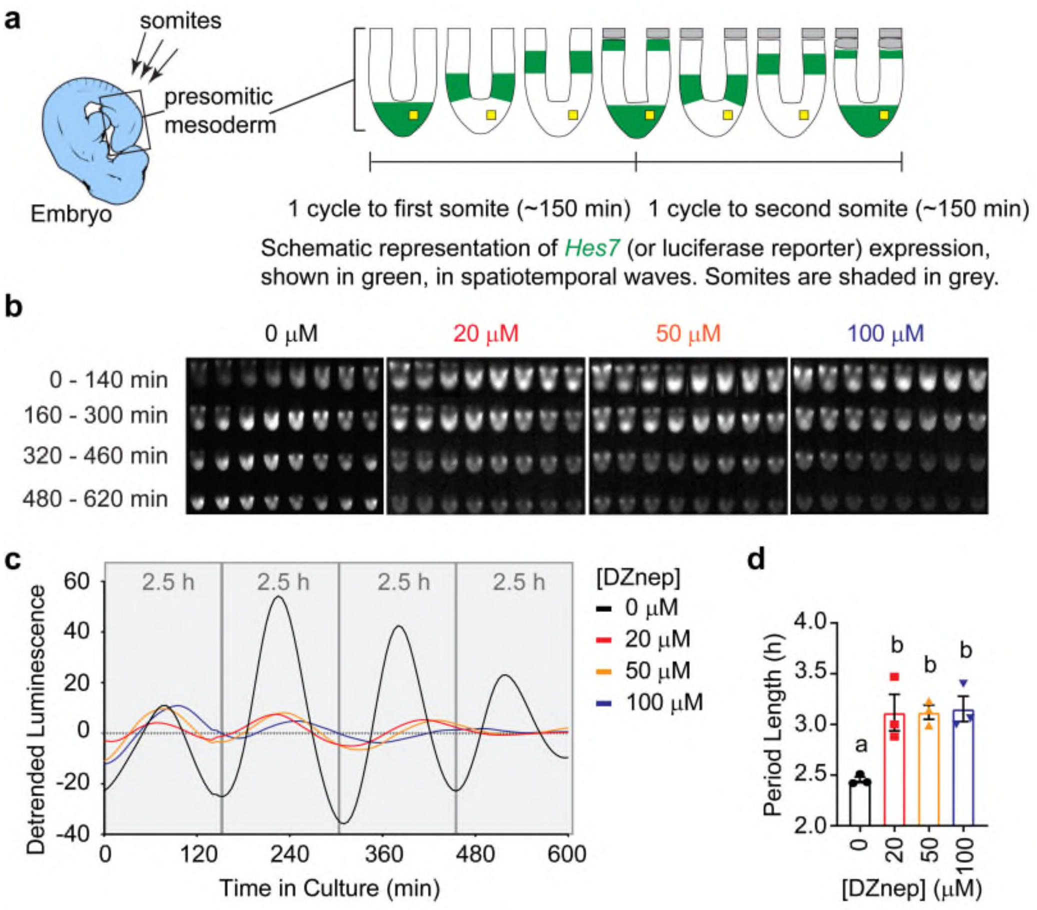


**Supplementary Figure 4: Somite segmentation rhythms are also sensitive to methylation deficiency.**
(**a**) Anatomic localization of the presomitic mesoderm in embryo. The yellow square defines the area from which luminescence was measured in (c). (**b**) Montage of luminescence time-lapse micrographs of one representative embryo for each treatment. One picture every 20 min is shown, starting from top left, each row corresponding to one *Hes7* expression cycle in the embryo treated with 0 μM DZnep. (**c**) Representative detrended luminescence measurements from one cultured embryo treated with various concentrations of DZnep as indicated. (**d**) Mean period ± SEM of n = 3 embryos per treatment, analyzed by One-Way ANOVA followed by Bonferroni's test; a vs. b, $p < 0.05$. Related to Figure 2.

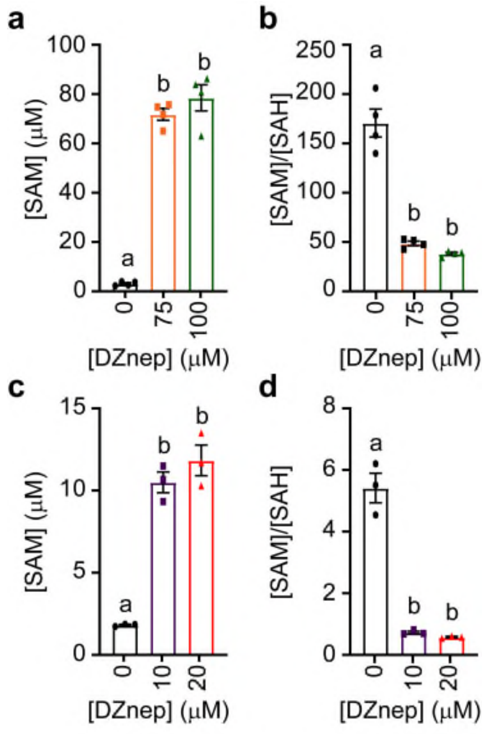


**Supplementary Figure 5: DZnep induces methyl potential collapse in unicellular green algae.** LC/MS/MS quantification of SAM (**a**) and calculation of the methylation potential ([SAM]/[SAH]) (**b**) in *C. reinhardtii* treated with the indicated concentrations of DZnep. LC/MS/MS quantification of SAM (**c**) and calculation of the methylation potential ([SAM]/[SAH]) (**d**) in *O. tauri* treated with the indicated concentrations of DZnep. Data show mean ± SEM, n = 4 wells for *C. reinhardtii* and 3 for *O. tauri*. All bar graphs analyzed by One-Way ANOVA followed by Bonferroni's test; all indicated comparisons (a vs. b) at least $p < 0.05$. Related to Figure 3.

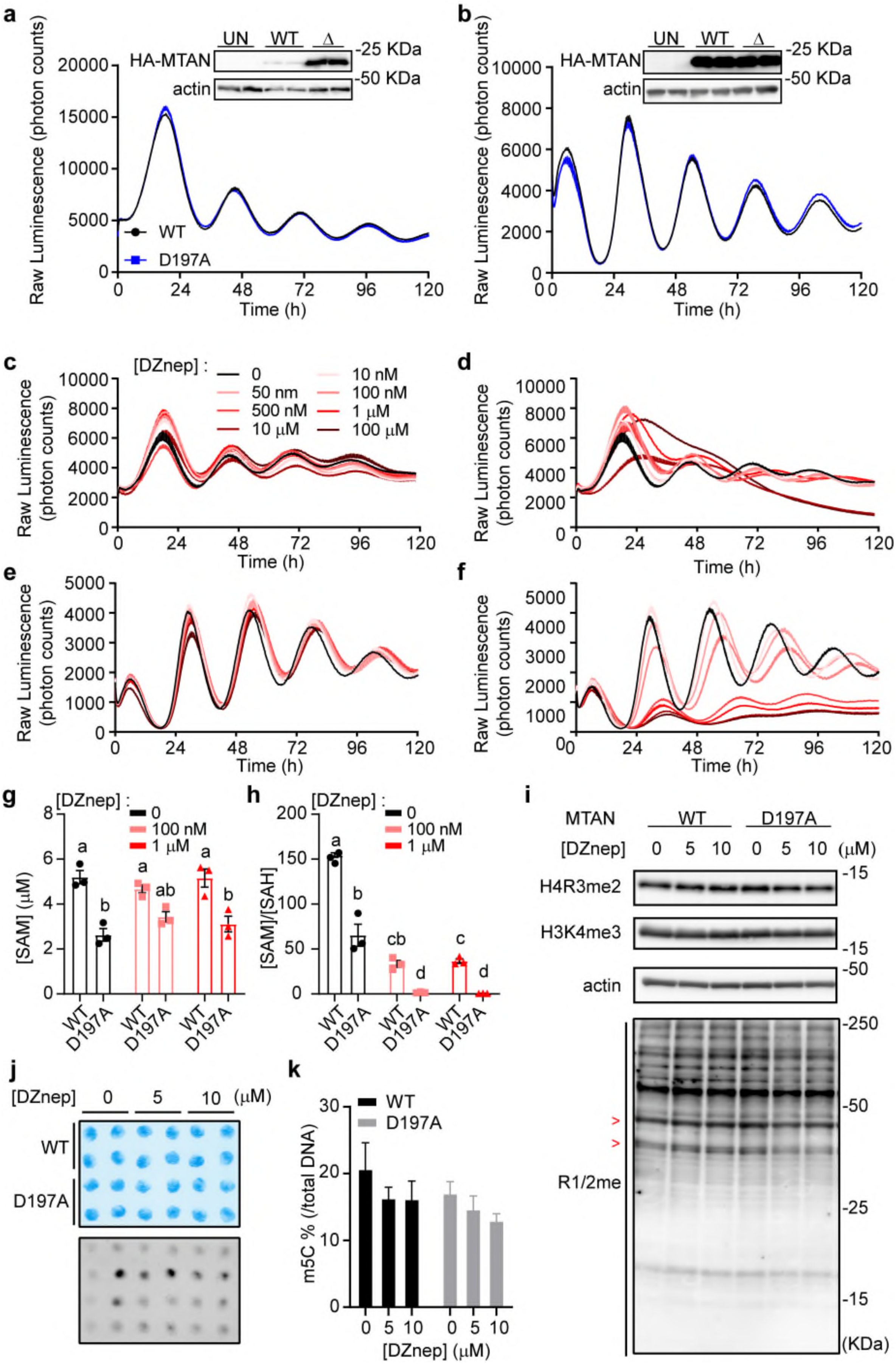
a
Raw Luminescence (photon counts)
HA-MTAN
actin
UN WT Δ
-25 KDa
-50 KDa
WT
D197A
Time (h)
b
c
[DZnep] :
0
10 nM
50 nm
100 nM
500 nM
1 μM
10 μM
100 μM
d
e
f
g
[SAM] (μM)
h
[SAM]/[SAH]
i
MTAN
WT
D197A
[DZnep]
0 5 10 0 5 10 (μM)
H4R3me2
H3K4me3
actin
R1/2me
(KDa)
j
[DZnep]
0 5 10 (μM)
WT
D197A
k
m5C % (/total DNA)
[DZnep] (μM)

**Supplementary Figure 6: Rewiring the methyl cycle protects mammalian cells from methylation deficiency.**

Mean luminescence ± SEM of n = 3 dishes of human *Bmal1*-luc U-2 OS (**a**) and PER2::LUC MEFs (**b**) acutely transfected with WT or D197A mutant MTAN and treated with vehicle, from Fig. 5. Immunoblotting results at the top confirm expression of WT and D197A MTAN in these cells. (**c**), Mean luminescence ± SEM of actively dividing *Bmal1*-luc U-2 OS cells (n = 3 dishes) acutely transfected with WT MTAN and treated with increasing concentrations of DZnep as indicated above the graph. (**d**), same as (c) but cells were acutely transfected with D197A MTAN. (**e**), Mean luminescence ± SEM of actively dividing PER2::LUC MEFs (n = 3 dishes) acutely transfected with WT MTAN and treated with increasing concentrations of DZnep as in (c). (**f**), same as (e) but cells were acutely transfected with D197A MTAN. (**g**), LC/MS/MS quantification of SAM and calculation of the methylation potential ([SAM]/[SAH], **h**) in DZnep-treated PER2::LUC MEFs transfected with the WT or D197A mutant MTAN, data shown mean ± SEM of n = 3 dishes, analyzed by Two-Way ANOVA followed by Bonferroni's test; all indicated comparisons (a vs. b vs. c vs. d) at least $p < 0.05$. (**i**) Immunoblots for Histone 4 Arginine 3 symmetric demethylation (H4R3me2), Histone H3 Lysine 4 trimethylation (H3K4me3), actin as a loading control, and monomethylated/dimethylated arginine (R1/2me) show mild methylation inhibition after 48 hours of DZnep treatment in PER2::LUC MEFs at the indicated concentrations, rescued by WT but not D197A MTAN. (**j**) Dotblot assay with anti-m6A antibody on total RNA extracted from PER2::LUC MEFs (n = 4 dishes) treated with the indicated concentration of DZnep for 48 hours shows no obvious methylation inhibition or effect of MTANs. The total RNA on the membrane (1200 ng/dot) was first stained with methylene blue as a loading control (top membrane). (**k**) ELISA-based quantification of global m5C DNA methylation in PER2::LUC MEFs treated as in (j). See also Figure 5.

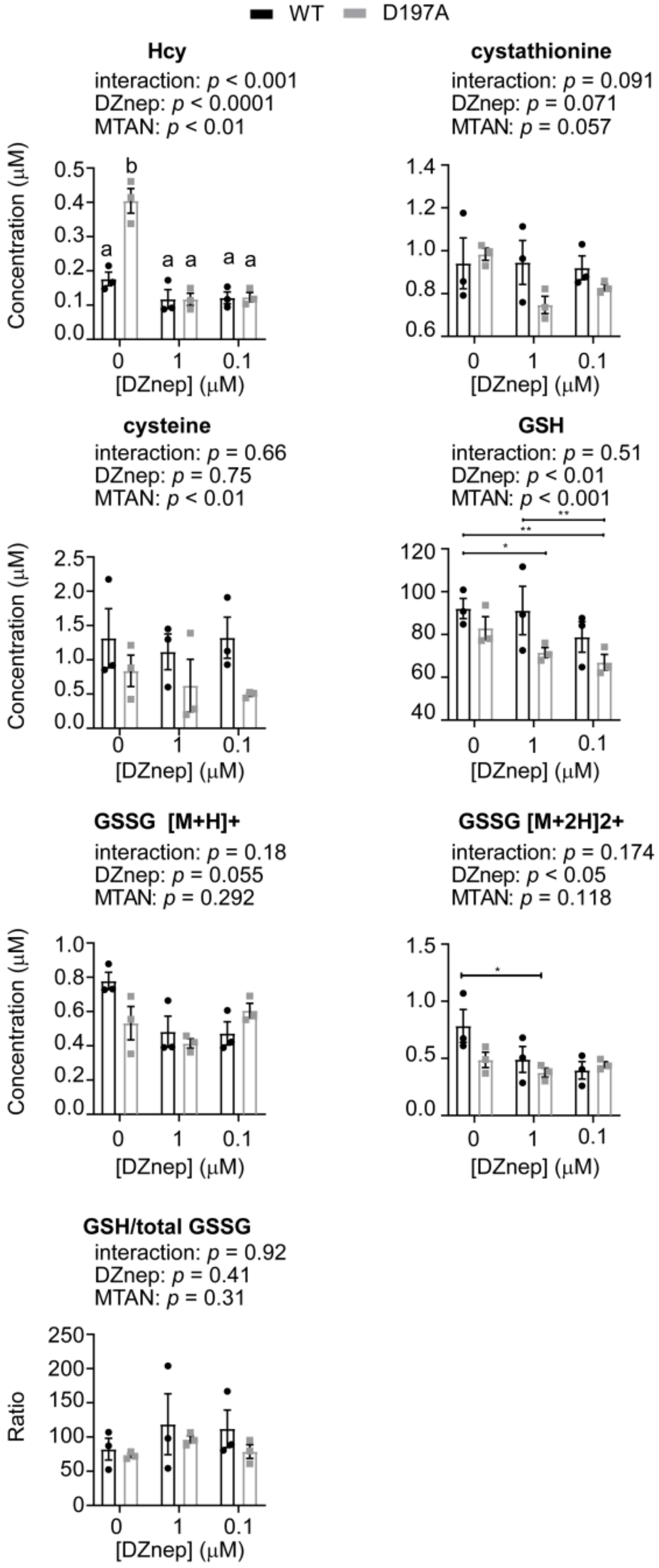
WT D197A
Hcy
interaction: p < 0.001
DZnep: p < 0.0001
MTAN: p < 0.01
cystathionine
interaction: p = 0.091
DZnep: p = 0.071
MTAN: p = 0.057
cysteine
interaction: p = 0.66
DZnep: p = 0.75
MTAN: p < 0.01
GSH
interaction: p = 0.51
DZnep: p < 0.01
MTAN: p < 0.001
GSSG [M+H]+
interaction: p = 0.18
DZnep: p = 0.055
MTAN: p = 0.292
GSSG [M+2H]2+
interaction: p = 0.174
DZnep: p < 0.05
MTAN: p = 0.118
GSH/total GSSG
interaction: p = 0.92
DZnep: p = 0.41
MTAN: p = 0.31
Concentration (μM)
Ratio
[DZnep] (μM)

**Supplementary Figure 7: Effects of MTAN and DZnep on the transulfuration pathway.**

LC/MS/MS quantification of transulfuration pathway metabolites, extracted from the same cells used in Fig. 5g. All graphs show mean ± SEM of n = 3 dishes, 3 technical replicates, analyzed by Two-Way ANOVA followed by Bonferroni's post-hoc test; *, $p < 0.05$; **, $p < 0.01$; a vs. b, $p < 0.0001$. Related to Figure 5.
