# Supplementary material for: Methylation deficiency disrupts biological rhythms from bacteria to humans
Source: Commun Biol. 2020 May 6;3:211. doi: 10.1038/s42003-020-0942-0 (PMC7203018; doi:10.1038/s42003-020-0942-0)
Supplement: Supplementary file 2 — Description of Additional Supplementary Files [file 42003_2020_942_MOESM2_ESM.pdf]

Description of Additional Supplementary Files

File Name: **Supplementary Movie 1**
Description: Animated structural superposition of AHCY from the 9 organisms investigated here, using human (1LI4), mouse (5AXA) or lupin (3OND) crystal structures as templates. The blue loop is specific to plants and green algae; DZnep is shown in yellow, NAD+ in grey.

File Name: **Supplementary Movie 2**
Description: Time-lapse luminescence recordings of one representative embryo for each treatment.

File Name: **Supplementary Movie 3**
Description: Time-lapse luminescence recordings of one representative embryo for each treatment, with luminescence shown as a pseudo-color green and merged with brightfield micrographs. The red arrows indicate the appearance of new somites.

File Name: **Supplementary Data 1**
Description: Raw luminescence data and other quantifications related to all main figures.
