# Supplementary material for: Methylation deficiency disrupts biological rhythms from bacteria to humans
Source: Commun Biol. 2020 May 6;3:211. doi: 10.1038/s42003-020-0942-0 (PMC7203018; doi:10.1038/s42003-020-0942-0)
Supplement: Supplementary file 7 — Reporting Summary [file 42003_2020_942_MOESM7_ESM.pdf]

nature research

Corresponding author(s): Jean-Michel Fustin
Hitoshi Okamura

Last updated by author(s): Mar 16, 2020

Reporting Summary

Nature Research wishes to improve the reproducibility of the work that we publish. This form provides structure for consistency and transparency in reporting. For further information on Nature Research policies, see Authors & Referees and the Editorial Policy Checklist.

Statistics

For all statistical analyses, confirm that the following items are present in the figure legend, table legend, main text, or Methods section.

n/a	Confirmed	
☐	☒	The exact sample size (*n*) for each experimental group/condition, given as a discrete number and unit of measurement
☐	☒	A statement on whether measurements were taken from distinct samples or whether the same sample was measured repeatedly
☐	☒	The statistical test(s) used AND whether they are one- or two-sided<br>*Only common tests should be described solely by name; describe more complex techniques in the Methods section.*
☐	☒	A description of all covariates tested
☐	☒	A description of any assumptions or corrections, such as tests of normality and adjustment for multiple comparisons
☐	☒	A full description of the statistical parameters including central tendency (e.g. means) or other basic estimates (e.g. regression coefficient) AND variation (e.g. standard deviation) or associated estimates of uncertainty (e.g. confidence intervals)
☐	☒	For null hypothesis testing, the test statistic (e.g. *F*, *t*, *r*) with confidence intervals, effect sizes, degrees of freedom and *P* value noted<br>*Give P values as exact values whenever suitable.*
☐	☒	For Bayesian analysis, information on the choice of priors and Markov chain Monte Carlo settings
☐	☒	For hierarchical and complex designs, identification of the appropriate level for tests and full reporting of outcomes
☐	☒	Estimates of effect sizes (e.g. Cohen's *d*, Pearson's *r*), indicating how they were calculated

Our web collection on statistics for biologists contains articles on many of the points above.

Software and code

Policy information about availability of computer code

Data collection	All PDB structures underwent preprocessing including the addition of missing amino acid atoms and hydrogens, removal of solvent atoms, and hydrogen-bond network optimization and residue protonation based on predicted pKa values using the Protein Preparation Wizard 51 of Maestro Schrödinger Release 2018-1 [Maestro, Schrödinger, LLC, New York, NY, 2018].<br>Homology models were constructed via the SWISS-MODEL server.<br>GROMACS version 2018 was utilised for energy minimization of all nine protein structures.<br>Molecular docking simulations were performed using using the Lamarckian Genetic Algorithm provided by the AutoDock4.2 suite.<br>Protein-ligand interactions were analysed in LigandScout version 4.2.<br>For the molecular structure of drugs shown in Fig. 4, the Open Source web application MolView was used.
Data analysis	All period estimations shown in the paper were performed by the circadian period analysis BioDare2, available online at https://biodare2.ed.ac.uk/.<br>Statistical analyses were performed with GraphPad Prism version 8.3.1.<br>A custom MATLAB script (Goya et al., 2016; available on request) was used to analyse the raw luminescence data from single C. elegans.

For manuscripts utilizing custom algorithms or software that are central to the research but not yet described in published literature, software must be made available to editors/reviewers. We strongly encourage code deposition in a community repository (e.g. GitHub). See the Nature Research guidelines for submitting code & software for further information.

Data

Policy information about availability of data

All manuscripts must include a data availability statement. This statement should provide the following information, where applicable:

- Accession codes, unique identifiers, or web links for publicly available datasets
- A list of figures that have associated raw data
- A description of any restrictions on data availability

All data generated or analysed during this study are included in this published article (and its supplementary information files).

Field-specific reporting

Please select the one below that is the best fit for your research. If you are not sure, read the appropriate sections before making your selection.

☒ Life sciences ☐ Behavioural & social sciences ☐ Ecological, evolutionary & environmental sciences

For a reference copy of the document with all sections, see nature.com/documents/nr-reporting-summary-flat.pdf

Life sciences study design

All studies must disclose on these points even when the disclosure is negative.

Sample size	No statistical methods were used to predetermine sample size.
Data exclusions	No data were excluded from the data shown.
Replication	All experiments were performed multiple times, data shown in the manuscript being a final representative replicate experiment.
Randomization	The experiments were not randomized.
Blinding	Investigators were not blinded to allocation during experiments and outcome assessment.

Reporting for specific materials, systems and methods

We require information from authors about some types of materials, experimental systems and methods used in many studies. Here, indicate whether each material, system or method listed is relevant to your study. If you are not sure if a list item applies to your research, read the appropriate section before selecting a response.

Materials & experimental systems

n/a	Involved in the study	
☐	☒	Antibodies
☐	☒	Eukaryotic cell lines
☒	☐	Palaeontology
☐	☒	Animals and other organisms
☒	☐	Human research participants
☒	☐	Clinical data

Methods

n/a	Involved in the study	
☒	☐	ChIP-seq
☒	☐	Flow cytometry
☒	☐	MRI-based neuroimaging

Antibodies

Antibodies used	Roche 11867423001 (Lot 10768600) for anti-HA antibody<br>Abcam ab23366 (Lot GR3223630-1) for mono- and di- methylated lysine<br>Abcam ab5823 (Lot GR3277687-1) for histone 4 Arginine 3 symmetric demethylation<br>Abcam ab8580 (Lot GR65362-2) for Histone H3 Lysine 4 trimethylation<br>Abcam ab412 clone 7E6 (Lot GR3211030-2) for monomethylated/dimethylated arginine<br>SySy 202 003 (Lot 202003/2-81) for m6A
Validation	All these antibodies have been well characterized and have been used in many publications, as reported by their respective companies website.

Eukaryotic cell lines

Policy information about cell lines

Cell line source(s)	Human U-2 OS cells stably transfected with a Bmal1-luciferase reporter vector (Baggs et al., 2009)<br>Mouse PER2::LUC MEFs cell lines (Yoo et al., 2004)<br>Per1b-luciferase cell line from zebrafish PAC2 cells (Vallone et al., 2004)<br>Ostreococcus tauri cells transgenically expressing a translational fusion of CCA1 to luciferase from the CCA1 promoter (Corellou et al., 2009)<br>Chlamydomonas reinhardtii strain CBR carrying a codon-adapted luciferase reporter driven by the tufA promoter in the chloroplast genome (Hwang et al., 1996)
Authentication	None of these cells were authenticated, nor was there any use for as they were received or generated from the original source.
Mycoplasma contamination	All cell lines tested negative for Mycoplasma contamination.
Commonly misidentified lines<br>(See ICLAC register)	None used

Animals and other organisms

Policy information about studies involving animals; ARRIVE guidelines recommended for reporting animal research

Laboratory animals	Transgenic ptim-TIM-LUC flies (Lamba et al., 2018)<br>C. elegans strain N2 (Bristol strain, wild-type was provided by the Caenorhabditis Genetics Center, University of Minnesota (cbs.umn.edu/cgc/home)<br>Synechococcus elongatus PCC 7942 reporter strain kaiBCp::luxAB71 (Xu et al., 2003)
Wild animals	No wild animals used
Field-collected samples	No field-collected samples used
Ethics oversight	No ethical approval was required as no animals/experiments subject to such approval was used in this study.

Note that full information on the approval of the study protocol must also be provided in the manuscript.
